# Sleep Deprivation Impairs Spatial Retrieval but Not Spatial Learning in the Non-Human Primate Grey Mouse Lemur

**DOI:** 10.1371/journal.pone.0064493

**Published:** 2013-05-22

**Authors:** Anisur Rahman, Solène Languille, Yves Lamberty, Claudio Babiloni, Martine Perret, Regis Bordet, Olivier J. Blin, Tom Jacob, Alexandra Auffret, Esther Schenker, Jill Richardson, Fabien Pifferi, Fabienne Aujard

**Affiliations:** 1 Mécanismes Adaptatifs et Evolution, UMR 7179 Centre National de la Recherche Scientifique, Muséum National d'Histoire Naturelle, Brunoy, France; 2 UCB Pharma s.a., Neuroscience Therapeutic Area, Braine l'Alleud, Belgium; 3 Department of Clinical and Experimental Medicine, University of Foggia, Foggia, Italy, and IRCCS San Raffalele Pisana, Rome, Italy; 4 Département de Pharmacologie Médicale, EA 1046, Université Lille Nord de France, UDSL, Faculté de Médecine, CHU, Lille, France; 5 CPCET-CIC, AP-HM Timone, INT, UMR 7289, CNRS - Aix Marseille Université, Marseille, France; 6 Johnson and Johnson Pharmaceutical Research and Development, A Division of Janssen Pharmaceutica, Beerse, Belgium; 7 Institut de Recherches Servier, Croissy-sur-Seine, France; 8 GlaxoSmithKline, R&D China U.K. Group, Stevenage, Hertfordshire, United Kingdom; Alexander Flemming Biomedical Sciences Research Center, Greece

## Abstract

A bulk of studies in rodents and humans suggest that sleep facilitates different phases of learning and memory process, while sleep deprivation (SD) impairs these processes. Here we tested the hypothesis that SD could alter spatial learning and memory processing in a non-human primate, the grey mouse lemur (*Microcebus murinus*), which is an interesting model of aging and Alzheimer's disease (AD). Two sets of experiments were performed. In a first set of experiments, we investigated the effects of SD on spatial learning and memory retrieval after one day of training in a circular platform task. Eleven male mouse lemurs aged between 2 to 3 years were tested in three different conditions: without SD as a baseline reference, 8 h of SD before the training and 8 h of SD before the testing. The SD was confirmed by electroencephalographic recordings. Results showed no effect of SD on learning when SD was applied before the training. When the SD was applied before the testing, it induced an increase of the amount of errors and of the latency prior to reach the target. In a second set of experiments, we tested the effect of 8 h of SD on spatial memory retrieval after 3 days of training. Twenty male mouse lemurs aged between 2 to 3 years were tested in this set of experiments. In this condition, the SD did not affect memory retrieval. This is the first study that documents the disruptive effects of the SD on spatial memory retrieval in this primate which may serve as a new validated challenge to investigate the effects of new compounds along physiological and pathological aging.

## Introduction

Sleep is an essential component of our life and is essential for maintaining normal daily life activities and key physiological functions such as thermoregulation [1], immune defense [2], energy conservation [3], tissue restoration [4] and brain plasticity [5]. Among these, sleep and brain functions are so intensely regulated that even a single night of SD in humans could induce mood disturbance, fatigue, daytime lethargy and several forms of cognitive impairment [6]. A bulk of studies has shown a strong correlation between SD and memory impairment in humans and rodents [7]–[9]. However, the role of sleep and impact of SD on memory processes i.e., acquisition, consolidation and retrieval is quite complex and seems to depend on the nature of the task used [10], [11]. There are two main methods to induce SD, the so called paradoxical SD procedure [12], [13] and the total SD procedure, which affects paradoxical and slow wave sleep [11]. In animal studies, paradoxical SD is usually obtained by placing the animal on a small platform surrounded by water [14], [15]. This type of manipulation has been noted to induce memory deficits in several tasks such as inhibitory avoidance [16], Morris water maze [17], [18], radial arm maze [19] and plus-maze discriminative avoidance tasks [13], [20]. In animal studies, total SD is simply achieved by removing or introducing objects within the cages [21] or by gently handling the animals to keep them awake [22]. This procedure has been shown to induce memory retrieval deficits in contextual fear conditioning, inhibitory avoidance and plus-maze discriminative avoidance tasks [23].

An important factor in SD studies is to determine when and how long the SD should be introduced in a specific sleep cycle. A leading hypothesis postulates that post-training sleep is required within a specific time window [14], [19], [24] following the training in order to facilitate consolidation of newly acquired information into long-term memory [25], [26]. In line with this hypothesis, SD after a learning task has been shown to result in subsequent memory deficits in both humans [27], [28] and rodents [29]. In humans, post-training sleep has been shown to modulate both spatial and contextual memories acquired during virtual navigation [27], [30], whereas post-training SD caused a disruption of spatial memory [9]. In rodents, SD immediately following the training has been shown to disrupt spatial reference memory in the radial arm maze [19] and in the Morris water maze [31]. In contrast, delayed SD (more than 4 hours) after the training appeared not to alter memory as shown in object recognition [32] and spatial memory paradigms [31], [33]. Altogether, these data suggest that sleep is important immediately after the training to properly encode and store traces in long-term memory for its subsequent retrieval. Furthermore, sleep may have a beneficial role before the training to prepare the brain for the acquisition of new information. Like the post-training SD, pre-training SD can affect the acquisition and memory retention in both humans [34] and rodents [13], [16], [35]–[38]. Specifically, several rodent studies have reported that pre-training SD affects conditioned aversion [35], avoidance learning [13], [16], [36], [37] and contextual fear conditioning [38]. Studies related to the impact of SD on spatial learning have delivered mixed results. One study has shown that pre-training SD can slow acquisition in Morris water maze [39], whereas other studies found no effect of SD on spatial learning [40], [41]. Finally, few studies investigated the impact of the pre-retrieval SD on memory retrieval showing that the ability to recall information is sleep-dependent [13], [20], and [23].

The interest of experimental models based on SD is not limited to basic research. Alzheimer's disease (AD), which is the most common form of dementia, deeply impairs sleep architecture and learning/memory processes [42], [43]. Therefore, it is important to define experimental “challenges” inducing some comparable features of AD in animal models in order to investigate disease mechanisms and evaluate therapeutic strategies, be they symptomatic or disease-modifying. Within this framework, the above-mentioned studies on SD in rodent provided quite useful information that could be relevant for AD research but, ideally, these results should be confirmed in species phylogenetically more proximal to humans, particularly with regard to sleep-wake cycles. In this context, a non-human primate model may provide better predictive validity with regard to cognitive testing compared to rodent models. We therefore conducted a series of experiments aimed to induce reversible cognitive impairment in a non-human primate, namely the grey mouse lemur, by using a SD procedure as a “challenge” to mimic some cognitive symptoms observed in AD. Interestingly, the mouse lemur shows features resembling human cerebral aging [44] and some aged mouse lemurs spontaneously develop AD related neuropathologies [45]. Moreover, the sleep-wake rhythm of this species has been described recently using telemetric electroencephalography and is suitable for the study of SD [46]. Specifically, we subjected mouse lemurs to 8 h of total SD by “gentle handling” either before the training or before the testing in a spatial task, which was a modified version of the Barnes maze, adapted for mouse lemurs [47]. In a first set of experiments, we investigated the effects of 8 h of total SD on spatial learning and memory retrieval after one day of training. In a second set of experiments, we tested the effect of 8 h of total SD on spatial memory retrieval after 3 days of training. We hypothesize that short-term (8 h) total SD induces impairment of acquisition and retrieval of memory in mouse lemurs.

## Materials and Methods

### Ethics statement

All experiments were performed in accordance with the Principles of Laboratory Animal Care (National Institutes of Health publication 86-23, revised 1985) and the European Communities Council Directive (86/609/EEC). The research was conducted under the authorization number 91–305 from the “Direction Départementale de la Protection des Populations” and under the approval of the Cuvier Ethical Committee (Committee number 68 of the “*Comité National de Réflexion Ethique sur l'Expérimentation Animale*”) under the authorization number 68-018. In accordance with the recommendations of the Weatherall report, “The use of non-human primates in research”, special attention was paid to the welfare of the animals during this work to minimize nociception [48].

### Animals

Thirty-one male mouse lemurs (2 to 3 years old) were used in this experiment. They were born and raised in the laboratory breeding-colony of Brunoy (MNHN, France, license approval N° A91.114.1) from a stock originally derived from the south-western coast of Madagascar 45 years ago. The animals were disease free and the general condition of captivity was maintained under constant temperature of 24–26°C and relative humidity of 55%. Measured food and water were allocated to each animal. The daily food allocation consists of fresh banana, apple and a homemade milky mixture of cereals, eggs and milk. Mouse lemurs are nocturnal primate and their physical activities and behaviors are driven by photoperiodic seasonal rhythms. During the long-day photoperiod (summer-like photoperiod), they have increased locomotor activities and metabolic rates to prepare to engage in reproduction. Conversely, during the short-day photoperiod (winter-like photoperiod), they show less locomotor activities, reduced metabolic rate and increased food intake. Animals were kept in alternating 6-month period of long-days (light∶dark 14∶10) and short-days (light∶dark 10∶14). Both experiments were performed during the long-day season. Mouse lemurs were housed in individual cages enriched with tree branches and wooden nest.

### Experimental design

**Figure 1:**
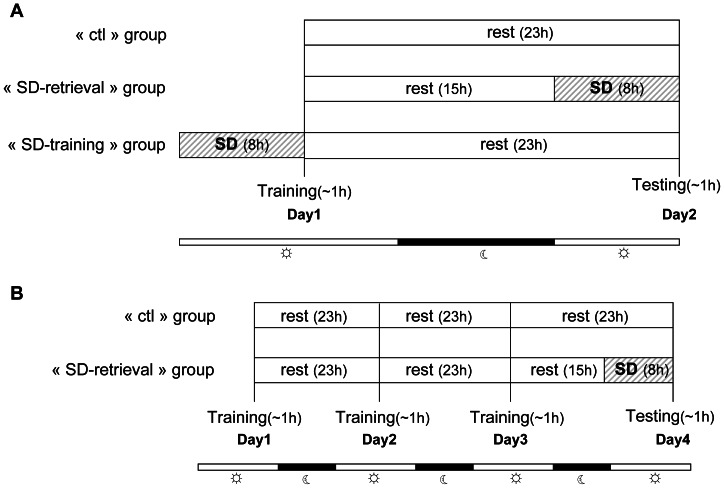
Experimental design. A) In experiment 1, animals of the “ctl” group received 23 h rest (light+dark phase) between the ends of the training on day 1 to the starting of the testing on the day 2. Animals of the “SD-retrieval” group received 15 h rest after training, followed by 8 h of sleep deprivation which occurred before testing. Animals of the “SD-training” group received 8 h of sleep deprivation before training, and then received 23 h rest before testing. B) In experiment 2, after 3 days of training, testing on day 4 was assessed after 8 h of SD in “SD-retrieval” group, or after rest in “ctl” group.

In experiment 1, we assessed and compared the effect of 8 h SD on spatial performances using a circular platform test. A total of 11 mouse lemurs went through 3 conditions of training and testing paradigm with 4 weeks of interval between each condition and the conditions were randomly assigned for each animal. In one condition, cognitive performance has been evaluated with training on day 1 and testing on day 2 without any SD to observe the baseline cognitive performance. In second condition, mouse lemurs were trained on day 1 and 8 h of total SD was performed on day 2 followed by a testing immediately after SD. In third condition, total SD was performed on day 1 followed by training and on day 2 animals underwent testing. Schematic presentation of the protocol has been depicted in detail in Figure 1A.

In experiment 2, we have increased the number of trials and days of training in order to assess the effect of 8 h total SD on long learning phase. A total of 20 new animals, 10 in each of “SD-retrieval” group and of “ctl” (control) group were used in this experiment. After 3 days of training, animals of the “SD-retrieval” group and of the “ctl” group were tested respectively after 8 h of total SD and without any SD. The protocol is elaborated in Figure 1B.

In all experiments, the interval between the training and testing trials was 24 h starting from the onset of training.

### Circular platform test

Spatial performances were assessed in a circular platform apparatus [47] which is a modified version of Barnes maze especially adapted for mouse lemurs. Briefly, the circular platform is divided into 12 quadrants with 12 equally spaced open circular holes (3 cm from perimeter) where a goal box can be affixed for the escape of the animal. The platform is fixed over a spring rotator so it could rotate freely in both directions, to avoid the use of intra-maze cues between successive trials. The whole platform is surrounded by a 15 cm high white wall with a transparent Plexiglas ceiling that allows the mouse lemur to see the extra-maze visual cues. The apparatus was surrounded by a black curtain hung from a square metallic frame, the ceiling of which is a one-way mirror to allow observation for the experimenter. A number of objects were attached along the inner surface of the frame to serve as visual cues. The starting box was an open-ended dark cylinder positioned in the center of the platform.

In all experiments, training trials (day 1) consisted of 4 trials of maximum 10 min, with an inter-trial interval of 5 min. During the first 2 trials, the animals were habituated in the maze with only one open compartment that contains the goal box and rests of the compartments were closed by thick white paper board. During the third and fourth trial, all the compartments were open and only one compartment gave access to the goal box (the target). Testing consisted of 2 trials of maximum 10 min, in the same condition as the last trials of the training day. Each trial started with the placement of animal in the starting box at the center of the maze. After 60 sec, the box was removed to release the animal. The aim of the tests was to reach the goal box positioned beneath one of the 12 compartments. The position of the target was fixed for each animal throughout one condition. In experiment 1, the position has been changed for subsequent conditions. When the animal reached the target, the trial was stopped and the animal was allowed to remain in the goal box for 2 min. Performance was assessed by the number of errors (entering the four limbs in an incorrect compartment), the rank of the target zone (two adjacent quadrants surrounding either side of the goal-box containing quadrant), and the latency (the total time required by the animal to reach the target) during the testing.

### Sleep deprivation

Mouse lemurs were subjected to 8 h SD (0–8) starting at the onset of light period (usual resting phase). The total SD was carried out in the first part of the light period because the sleep is at its maximum during this period [46]. During the whole SD period, mouse lemurs were under constant visual observation in their home cage. The nest and the tree branches were removed from the cage for proper visualization of the animals. SD was achieved by gentle handling which consists of a standardized procedure of tapping on the cage, moving the index finger in front of the cage and gently shaking the cage if required. If the animal sits more than five minutes in a place without any activities they were re-located. When the above measures were not sufficient to keep the animals awake the front door was opened and closed to stimulate the animals. To confirm the total SD, four animals were under telemetric electroencephalogram (EEG) and electromyogram (EMG) recordings during the SD. To avoid any impact of implantation on behavioral tests, surgical procedure was performed on independent group of animals.

### EEG and EMG recordings

Wireless telemetry transmitter weighing 2.5 g (PhysioTel F20-EET; Data Science, St Paul, MN, USA) was implanted in the abdominal cavity under intraperitoneal ketamine anesthesia (100 mg/kg; Imalgene, Merial, 69007, Lyon-France). The transmitter is equipped with the simultaneous recording for one EEG and one EMG channel (1–500 Hz sampling rate). The electrode leads were threaded subcutaneously from the abdomen to the skull incision. For EEG recording, the electrodes were fixed to the skull using dental cement over the anterior frontal cortex according to the stereotaxic atlas of the mouse lemur brain [49]. For EMG recording, a pair of bipolar electrodes was sutured in the neck muscles with nonabsorbable polyamide suture. Animals were allowed to recover at least two weeks prior to the start of the experiment. EEG and EMG data were continuously collected using PC running Dataquest software (Data Science International, St Paul, MN, USA). A receiver base (RPC-1, Data Science) that relayed transmitter data to the PC was placed on the floor of the home cage inhabited by the implanted animals. For each implanted animal a continuous 3 d baseline recording was obtained prior to the recoding of SD time-period. The EEG data was analyzed by Neuroscore v 2.1.0 (Data Science International, St Paul, MN, USA). The data of SD period was compared with the data of resting phase (before SD) corresponding to SD schedule (0–8 h).

### Statistical analysis

For all statistical assessments, data were first assessed for normality using R 2.12.1 software. The Kruskal-Wallis (χ^2^-value) or Mann-Whitney (W-value) analyses were performed to compare the data between the control and SD groups, respectively for experiment 1 and experiment 2. Effect of day was evaluated by paired Wilcoxon signed rank test (V-value). A *P*-value of <0.05 was considered significant. All values are given as median and interquartile (IQ: lower quartile-upper quartile) in text and are represented in box plot in figures.

## Results

### Electroencephalogram findings

**Figure 2:**
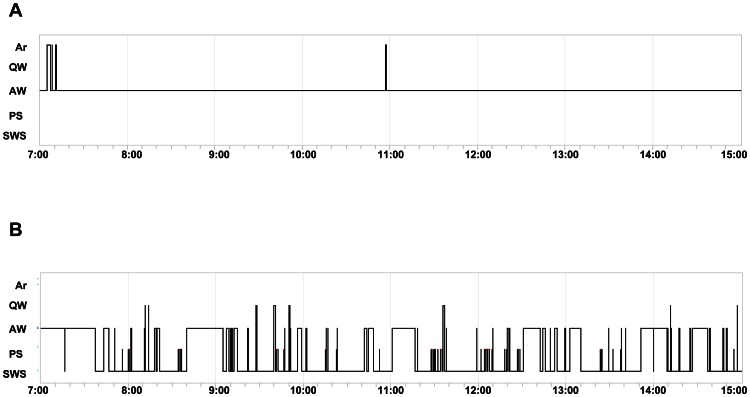
Hypnograms recorded during A) 8 h of SD period and B) corresponding 8 h of control period by electroencephalography. Ar = artifact, QW = quiet wake, AW = active wake, PS = paradoxical sleep, SWS = slow wave sleep.

We first recorded the 3 days of baseline sleep-wake cycle parameters including the daily profile of slow wave sleep (SWS), paradoxical sleep (PS), active wake (AW) and quiet wake (QW). The sleep deprived animals passed through the following sleep-wake cycle during 8 h of SD (AW = 97.16%±2.38; QW = 0.06%±0.11; PS = 0%; SWS = 0%; and Artifact = 2.76%±2.27; Figure 2A) as compared to the corresponding control phase of EEG (AW = 32.66±14.86; QW = 3.86±6.18; PS = 5.06±6.86; SWS = 53.26±9.33; and Artifact = 5.13±5; Figure 2B). Sleep-deprived animals showed 0% of sleep (SWS+PS) during 8 h of total SD as compared with 58% of sleep (SWS+PS) during the control phase. These data proved the robustness of our total SD procedure.

### Experiment 1

We first tested the impact of 8 h total SD on spatial learning and memory retrieval after one day of training in a circular platform task. Mouse lemurs (n = 11) were subjected to three conditions: i) the animals of “ctl” (control) group were trained on day 1 and tested on day 2 without any SD, ii) the “SD-training” group animals were sleep deprived for 8 h just before training on day 1 and tested on day 2 and iii) the “SD-retrieval” group animals were trained on day 1 and sleep deprived for 8 h just before testing on day 2.

In the day 1 trials (training day), no differences were found among conditions in the number of errors, in the number of repetitions, in the rank of the zone (the zone corresponded to the target and the two adjacent compartments) and in the latency to reach the target (χ^2^ = 2.48; 0.31; 0.35; 3.67 respectively; p>0.2).

**Figure 3:**
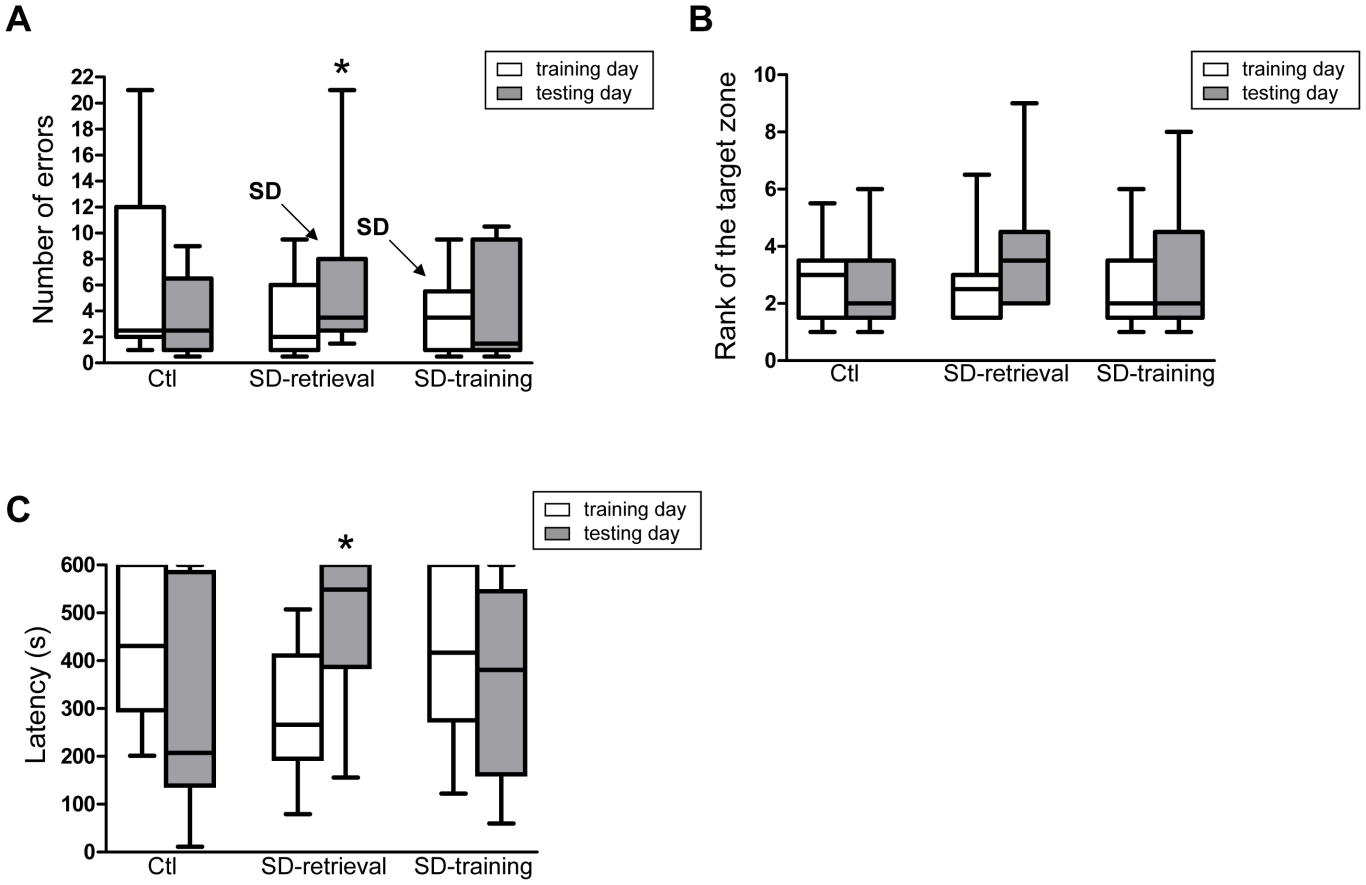
Effects of sleep deprivation on spatial learning and memory after 1 day of training A) Number of errors, B) Rank of the target zone and C) Latency (sec). Performances during training (day 1) and testing (day 2) in “ctl”, “SD-retrieval” and “SD-training” groups of the experiment 1. Significant differences for the comparison of day 1 and day 2 (Wilcoxon signed rank test) are indicated as * (p<0.05).

The number of errors during day 2 trials (testing day) was significantly higher than during day 1 trials in the “SD-retrieval” group (median: 2, IQ: 1–5.5 for day 1; median: 3.5, IQ: 2.5–7.8 for day 2; V = 10; p = 0.045; Figure 3A). No significant differences were observed for the rank of the zone between day 1 and day 2 (V = 20.5; p = 0.28; Figure 3B), indicating that the impairment in the number of errors before reaching the target was selective to a spatial memory deficit. Moreover, the pre-retrieval sleep-deprived animals (“SD-retrieval” group) took more time to reach the target after the SD (median: 266 s, IQ: 204.3–388 s for day 1; median: 548.5 s, IQ: 388.5–600 s for day 2; V = 10; p = 0.042; Figure 3C). These animals showed a tendency to increase the number of repetitions between day 1 (median: 0, IQ: 0–0.8) and day 2 (median: 0.5, IQ: 0–2.3; V = 2.5; p = 0.06). Altogether, these results indicated that 8 h SD impaired spatial memory retrieval after one day of training.

In contrast to the “SD-retrieval” group, control animals (“ctl” group) and pre-training sleep-deprived animals (“SD-training” group) did not show any differences between day 1 and day 2 trials with respect to the number of errors, the number of repetitions, the rank of the zone and the latency (p>0.2; Figure 3A–C). These results indicated that SD had no effect on spatial learning.

### Experiment 2

In this experiment, we investigated the impact of 8 h total SD on spatial memory retrieval after an increased amount of days of training and of trials during 3 consecutive days. Mouse lemurs were subjected to two conditions: i) “ctl” animals (n = 10) were trained on day 1, day 2, day 3 and tested on day 4 without any SD, ii) “SD-retrieval” group animals (n = 10) were trained on day 1, day 2, day 3 and sleep deprived for 8 h just before testing on day 4.

In the day 3 trials, no significant differences were found between the two groups in the number of errors, in the number of repetitions, in the rank of the zone and in the latency to reach the target (W = 39.5; 35.5; 51; 55 respectively; p>0.2).

**Figure 4:**
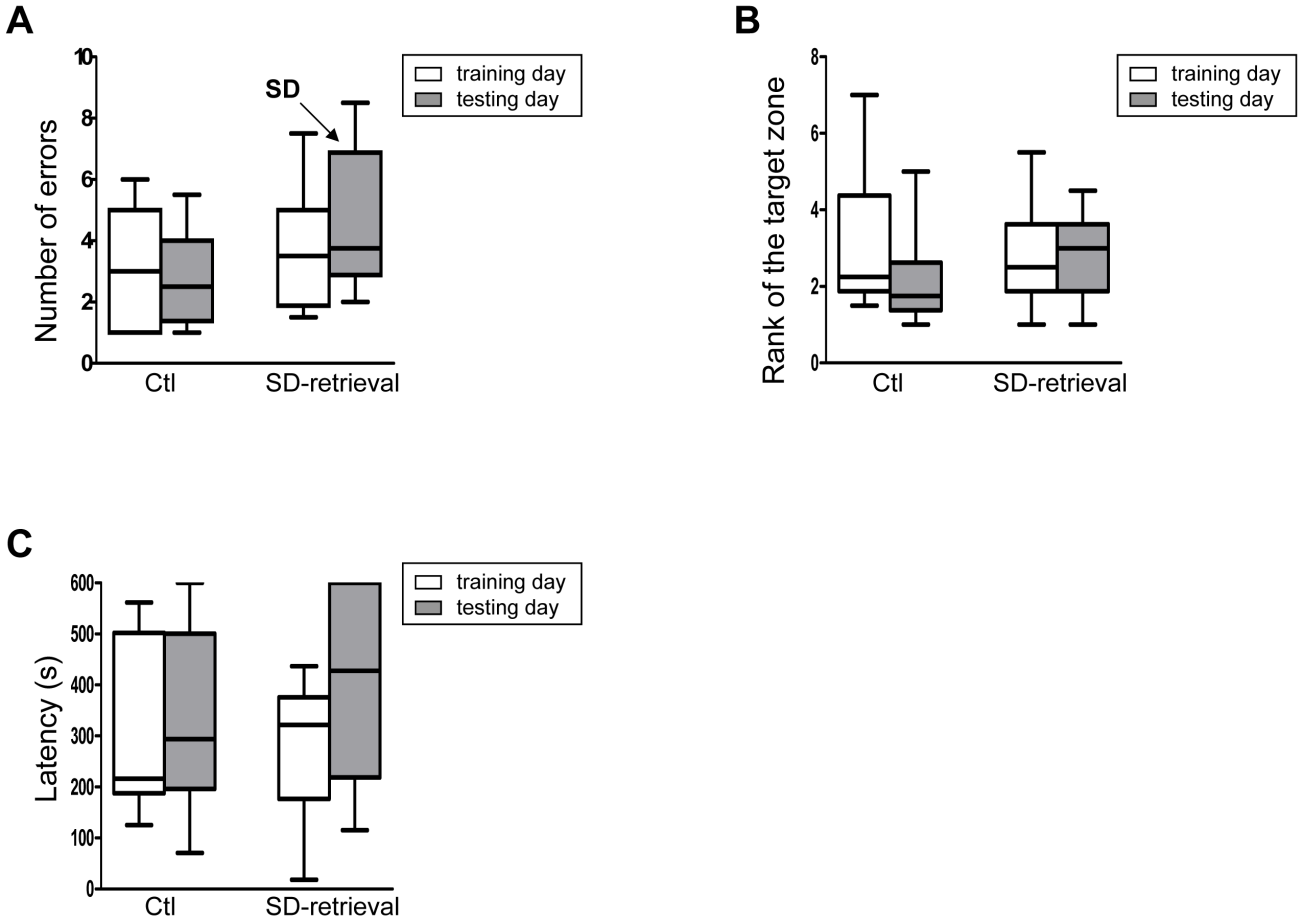
Effects of sleep deprivation on spatial learning and memory after 3 days of training (A) Number of errors, (B) Rank of the target zone and (C) Latency (sec). Performances during last training (day 3) and testing (day 4) in “ctl” and “SD-retrieval” groups of the experiment 2.

The number of errors, the number of repetitions, the rank of the target and the latency did not differ between the day 3 and day 4 trials in the “SD-retrieval” (V = 17.5; 19; 19; 12 respectively; p>0.2) and in the “ctl” group (V = 28; 15; 35.5; 28 respectively; p>0.2; Figure 4 A–C). These results indicated that the sleep deprivation had no effect on spatial memory retrieval after three days of training.

## Discussion

In this study, we showed that 8 h of total SD impaired the retrieval, but not the learning, of a one-day spatial memory task in mouse lemurs. Furthermore, retrieval memory was sensitive to the total SD after one day but not after three days of training. These findings suggest that the total SD acts differentially as a function of the memory processes (i.e. training vs. retrieval) as well as of the amount of trials and of the level of training. The successful development of neuropathological features of AD on rodent and invertebrate models by genetic manipulation or other means could not able to obviate the difficulties of modeling the disease manifestations that are uniquely seen in human. Moreover, behavioral paradigm cannot be convincingly ascertained in animal models and the issue becomes more complex when the animal is to be used for therapeutics. Although transgenic mouse models of AD show a number of neuropathological features of AD but their translational efficacy of drug testing is not satisfactory, whereas, old-world monkeys showed better predictive capability in cognition-enhancing drug research despite the absence of AD-like syndromes in most of the aged monkeys [50]. Merely comparable brain physiology of non-human primate with human allows for a greater level of face validity and predictability of therapeutic compounds in terms of clinical potency and efficacy as compared with lower species. Keeping these in mind, our goal was to create some cognitive impairment by SD challenge in this primate and to test the clinical efficacy of drugs by reversing those impairments.

In the experiment 1, pre-training SD animals demonstrated unaffected acquisition of the spatial task as compared with the control animals, suggesting that 8 h of total SD could not impair the spatial learning occurring during the training phase. Moreover, we showed no retention deficits at 24 h delay as a consequence of the pre-training SD. These results are in agreement with previous evidence in rats showing that 24 h of sleep disturbance (i.e. interruption) prior to a water maze training had no effect on the learning or later retention of the spatial location [40], [41]. Similarly, Leenaars et al. [51] have reported no effect of 12 h SD on spatial reversal learning using a skinner box. However, the impact of SD depends very much on the type of task used. Indeed, several studies showed that SD given prior to training impaired conditioned aversion [35], inhibitory avoidance learning [13], [16], [36], [37], [52] and contextual fear conditioning [38]. All these tests are aversively-motivated and are strongly influenced by anxiety and motivation [13], [52], [53]. For instance, Alvarenga et al. show that pre-training SD rats expressed anxiolytic- and mania-like behavior, which can explain the negative effect of SD on learning [13]. This suggests that the apparent discrepancies noted above might be due to interference by stress-related SD.

In the present study, the detrimental effects of the pre-testing SD in the spatial memory retrieval (after one day of training) are in line with previous rodent studies showing memory impairment induced by pre-testing SD in avoidance tasks [13], [20], [23]. However, these studies argued that this impairment induced by the SD was due to state-dependent learning, which postulates that animals need to be in the same physical/mental state (here under the “SD” state) during the learning and the retrieval phase. The present findings do not fit the hypothesis of state-dependency, at least in mouse lemurs: we did not find learning and retrieval deficits when SD was applied before the training (experiment 1) or before the testing after 3 days of training (experiment 2). Very few studies have investigated the effect of pre-retrieval SD on memory. Our findings highlight that a delayed SD (15 h after the training) can affect spatial memory retrieval. While this finding did not precisely define the time-window(s) after training during which the SD can impair the retrieval, our results could be explained by the consolidation hypothesis. This hypothesis postulates that neuronal circuits and activity pattern that are involved during the training undergo a post-processing during which the traces are reactivated, analyzed and gradually incorporated in to the brain's long-term event memory [54]–[58]. On the other hand, here we report that the impairment of memory retrieval induced by pre-testing SD depends on the training protocol: pre-testing SD impaired retrieval memory after one day of training (experiment 1), but not after three days (experiment 2) of training. This finding could be explained by the fact that the longer the period of training, the stronger the formation of the memory traces [59], [60].

Questions have been raised concerning the methodological issues of SD where stress is an inevitable confounding cofactor. Previous SD studies have adopted radically diverse methods ranging from flower pot to treadmill or mild stimulation to gentle handling for shorter period protocols. These different methodological interventions produce a different level of physical stress, motivation, locomotor activities and other variables. In this context, one would argue that SD might affect relevant behavioral parameters (number of errors and the latency in our circular platform task) by its effect on anxiety, motivation or motor activity. However, the present SD procedure is less stressful than that typically used with rodents (i.e. single/multiple platform or treadmill or novel object introduction). In the present study, we used the following procedure to minimize the level of stress during the experiments: i) mouse lemurs were sleep-deprived in their home cage, ii) the presence of the experimenter was an effective stimulant for the vigilance of these primates and iii) only a minimal stimulation was applied, especially at the final hours of the procedure. Finally, behavioral performances in the circular platform were not affected in all sleep-deprived animals, since the pre-training sleep-deprived animals of the experiment 1 and the pre-testing sleep-deprived animals of the experiment 2 showed a similar amount of errors and latency as compared with the control animals. All these arguments do not support a major impact of stress/anxiety in our experiment.

The present findings suggest that the pre-testing total SD selectively impaired the retrieval of spatial memory task. This effect seems to be dependent on the amount of days/trials of the training prior to the total SD. These findings extend previous evidence on the total SD effects on learning and memory and unveil, for the first time that sleep is involved in memory retrieval of a spatial task in a non-human primate. As mentioned above, the ultimate goal of the present study was to test a challenging procedure able to induce a reversible impairment of the memory function in a non-human primate. We are aware that the complex cognitive abilities impaired in AD patients are quite challenging to be mimicked in animal models. Nevertheless, all the available treatments of AD were preliminarily tested in rodents. Non-human primates have been used for decades for the development of cognition-enhancing pharmacological agents but it is only recently that a much greater appreciation of this translational applicability has emerged [50]. The present findings further motivate the use of mouse lemur as a promising animal model for the study of human aging and therapeutic programs [44].
